# African trypanosomes

**DOI:** 10.1186/s13071-019-3355-5

**Published:** 2019-04-29

**Authors:** Mathieu Cayla, Federico Rojas, Eleanor Silvester, Frank Venter, Keith R. Matthews

**Affiliations:** Institute for Immunology and Infection Research, School of Biological Sciences, University of Edinburgh, Edinburgh, UK

**Keywords:** *Trypanosoma brucei*, Sleeping sickness, Trypanosome

## Abstract

African trypanosomes cause human African trypanosomiasis and animal African trypanosomiasis. They are transmitted by tsetse flies in sub-Saharan Africa. Although most famous for their mechanisms of immune evasion by antigenic variation, there have been recent important studies that illuminate important aspects of the biology of these parasites both in their mammalian host and during passage through their tsetse fly vector. This Primer overviews current research themes focused on these parasites and discusses how these biological insights and the development of new technologies to interrogate gene function are being used in the search for new approaches to control the parasite. The new insights into the biology of trypanosomes in their host and vector highlight that we are in a ‘golden age’ of discovery for these fascinating parasites.

**Electronic supplementary material:**

The online version of this article (10.1186/s13071-019-3355-5) contains supplementary material, which is available to authorized users.

## What are trypanosomes?

The Trypanosomatidae (Phylum Euglenozoa) are obligate parasitic protozoans, which infect all vertebrate classes, in addition to insects and plants. Trypanosomatids are uniflagellated, and like other organisms in the order Kinetoplastida, are characterised by a modified mitochondrial genome known as the kinetoplast [1, 2]. This is composed of DNA ‘mini’- and ‘maxicircles’ [3, 4], which can vary in number and catenation depending on the particular species [5]. Among the trypanosomatids, *Trypanosoma* is a genus of particular medical and veterinary concern [6, 7]. The Salivaria group of trypanosomes, so named for being transmitted in the infected saliva of a tsetse fly vector (*Glossina* spp.), is represented by *Trypanosoma brucei*, *T. congolense* and *T. vivax*. The former is the most well-studied of the salivarian trypanosomes, with subspecies *T. b. gambiense* and *T. b. rhodesiense* being the causative agents of human African trypanosomiases (HAT). The dixenous life-cycle of *T. brucei* comprises vertebrate stages and tsetse stages, which involves differentiation through a number of morphological forms, accompanied by remodelling of gene expression and metabolic processes [8, 9] (Additional file 1). In the bloodstream, two morphotypes exist, slender and stumpy forms [10, 11]. Slender forms evade the mammalian antibody response through antigenic variation, entailing expression of a variable surface glycoprotein (VSG) monolayer which covers the trypanosome cell. Changes in the expression of VSG genes generate antigenically distinct subpopulations, which can evade the host immune response, and so sustain the parasite infection [12, 13]. The transition from slender to stumpy forms is regulated by a quorum sensing (QS) type process which prolongs host survival and promotes transmission because stumpy forms are non-proliferative in the blood, but preferentially survive in and colonise the tsetse midgut.

While *T. brucei* is certainly a model for trypanosome biology, and indeed that of other kinetoplastids, significant variation in morphology, transmission, and life-cycle, exists between different trypanosome species. Neither *T. vivax* nor *T. congolense*, for example, (both causing animal African trypanosomiasis, AAT), show morphological differentiation in the bloodstream, though both exhibit density-dependent growth control [14, 15]. Also, *T. b. equiperdum* has circumvented the need for an arthropod vector altogether, and instead is sexually transmitted between equines. Exciting advances in our understanding of trypanosome biology continue to be made, in addition to the development of new tools for studying these organisms. This Primer will highlight important recent advances in this field, as well as identifying areas that warrant investigation in future.

## Why study the biology of trypanosomes?

Trypanosomatids diverged from other eukaryotic lineages at least 500 million years ago and so provide a valuable model for evolutionarily distinct eukaryotic processes [16, 17]. Despite their divergence, they exhibit tractable and fascinating biology in terms of their nuclear and nucleosome architecture/substructures, transcription, organellar segregation, DNA replication, developmental biology and flagellar motility, for example [18, 19]. This tractability has been greatly assisted by the capacity to culture trypanosomatids, to generate different life-cycle stages and by the development of advanced genetic tools. Inducible systems for the regulated ectopic expression of genes, or RNAi mediated knockdown have been widely exploited, with CRISPR-Cas9 genome editing [20, 21] recently added to the already large toolbox available to manipulate trypanosomatid genomes and gene expression. As well as their intrinsically interesting biology, trypanosomes remain important causes of disease. Human cases have reduced in recent years, currently at approximately 2000 cases per year, although under-reporting and the discovery of asymptomatic carriers [22] may require revaluation of the prospects for disease elimination. For the animal disease, trypanosomosis remains prevalent and devastating, generating significant restrictions on animal productivity in the tsetse belt of sub-Saharan Africa.

The genome of *T. brucei* has 11 megabase-chromosomes (~of 35 Mb total) as well as 5 intermediate (200–300 kb) and ~60–100 mini-chromosomes (30–150 kb) [23–25]. The megabase chromosomes are segregated *via* a kinetochore machinery that appears highly divergent with respect to the canonical eukaryotic model [26]. Genes are organised and co-transcribed in large multi-gene (polycistronic) units [27]. There is little evidence of clustering of genes according to life-cycle regulation in *T. brucei,* such that post transcriptional mechanisms control gene expression (RNA stability, translation, codon usage) [18]. Most protein-coding genes are transcribed by RNA polymerase II (RNAP II) with the mRNA for all protein-coding genes being capped by addition *via trans* splicing of a 39 nt ‘spliced leader’ (SL) sequence, this assisting translation and stability. Only two genes have been identified with an intron that requires cis splicing [28]. The promoter for the SL array, consisting of 100–200 tandem SL RNA mini-exons [29], possesses a very divergent RNAP II initiation complex [30, 31]. Sites of RNA polymerase II initiation in the genome are epigenetically marked, being enriched in histone modifications H4K10ac and H3K4me3 and four different histone variants that are also found at convergent transcription strand switch regions [32].

*Trypanosoma brucei* also possesses a uniquely evolved multifunctional RNAP I system, responsible for the transcription of ribosomal gene units, as well as the major surface proteins procyclins (expressed on tsetse midgut forms) and bloodstream VSG genes [33, 34]. It is estimated that there are around 10 million copies of VSGs on the surface of a parasite shielding invariant surface proteins [35–37]. *Trypanosoma brucei* possesses ~2500 VSG genes or pseudogenes [24], located in subtelomeric regions of the chromosomes (comprising ~30% of the genome) [12, 38]. It is now understood that during the course of an infection there are up to 100 VSGs that are expressed in the population at a peak of infection with a few variants dominating at each peak, allowing immune evasion [13, 39]. In the bloodstream stage, there are ~15 telomeric VSG expression sites (ES), each containing 8–9 ES associated genes (ESAGs), 70 bp repeats and a VSG gene [40, 41]. *Trypanosoma brucei* exhibits monoallelic expression of one ES at a time allowing the expression of one VSG variant per parasite [34, 42, 43]. ES transcription does not occur in the nucleolus, unlike other RNAP I transcripts (rRNA, procyclin genes [44, 45]), but at an extranucleolar focus of RNAP I (the ‘expression site body’) [44, 46, 47]. The switch between different ES is controlled epigenetically [48] and occurs early during the time course of an infection [13, 39]. Later, but still early during the infection, DNA recombination of entire VSG genes occurs into the active ES, from mainly telomeric regions, and is mediated *via* the 70 bp repeats. When this repertoire is exhausted, recombination occurs from subtelomeric array VSGs, with segmental VSGs conversion producing mosaic VSGs [13, 23, 39, 41, 49–51]. These VSG recombinations are dependent on a DNA break [40].

Cytological organisation and flagellar motility have also proved fascinating areas of trypanosome biology of relevance across eukaryotes. Trypanosomes are highly ordered, with their single copy organelles precisely positioned and their movements carefully orchestrated during cell division [52]. Ultrastructural studies using electron microscopy and electron tomography have revealed how the microtubule corset of the trypanosome is co-ordinated with the parasite flagellar basal body and flagellum close to the flagellar pocket [53–56]. This organisation and the experimental tractability of trypanosomes has made these organisms invaluable as a model for flagellar and ciliary biology in eukaryotic organisms [57, 58].

## Three advances in the last decade

### High-throughput phenotyping

With the development of Illumina sequencing, a method to survey the representation of genetically distinct cells in a complex population was provided. This has proved immensely powerful in trypanosome research, allowing a genome-wide RNA interference-based phenotyping approach (RNAi target sequencing, RIT-seq) [59]. Using doxycycline-regulated RNAi induction, cytocidal or cytostatic phenotypes that diminish the representation of the RNAi-target sequence relative to the population, reveals “cold spots” that correspond to predicted mRNA sequences. Thus, by combining RNAi and Illumina sequencing, it has been possible to identify genes important for trypanosome growth in the bloodstream and procyclic form as well as during differentiation between these forms.

The use of RIT-seq was also applied in positive selective screens for the identification of genes that contribute to drug resistance [60]. Screens were performed using all current HAT drugs (Eflornithine, Suramin, Nifurtimox, Pentamidine and Melarsoprol) and each yielded a population of cells displaying an RNAi-inducible drug resistance phenotype, associated with the downregulation of specific genes. These have provided molecular insight into the trafficking pathways and resistance mechanisms for anti-trypanosomal drugs.

RIT-seq had also been used effectively to explore trypanosome biology, an example being its use to dissect the signal-response pathway that promotes stumpy formation [61]. Although the quorum sensing signal SIF (stumpy induction factor) was unidentified, the screen exploited the ability of cell-permeable analogues of cAMP or AMP to mimic SIF in laboratory adapted trypanosome lines [62]. By using cAMP analogue-induced cell cycle arrest as the selection, genes involved in the signal-response pathway that promotes stumpy formation were identified from the RNAi inserts enriched in outgrowing proliferative parasites.

### Environmental and cell-cell communication

Akin to many other single-celled microbes, it has become clear that African trypanosomes exhibit social behaviours throughout their life-cycle. One example is the coordinated swarming or social motility of *T. brucei* procyclic forms observed on culture plates [63, 64]. cAMP plays a role in this phenomenon, with knockdown of the cAMP-specific phosphodiesterase PDEB1 found to inhibit social motility [65], and knockdown of certain adenylate cyclases resulting in a ‘hypersocial’ phenotype [66]. This is not dissimilar to the role of cyclic-diGMP in bacterial swarming motility [67].

Social behavior in bloodstream *T. brucei* in the form of quorum sensing was described in the 1990s [10, 68], with the demonstration that differentiation from the slender to the stumpy bloodstream form occurred in a density-dependent manner resulting from accumulation of an unidentified parasite-derived factor. As detailed above, the signaling components and RNA regulators that drive the generation of the stumpy form, have begun to be revealed in the last decade [61]. Interestingly, despite their lack of morphological differentiation in the bloodstream, the signaling components required to perceive and respond to the density-signal in *T. brucei* are conserved in *T. congolense*. Moreover, *T. congolense* produces a QS signal that causes *T. brucei* to differentiate to stumpy forms in mixed infections [69], revealing that inter-species QS is possible, just as occurs in bacterial communities.

Consistent with the trypanosome’s reliance on post-transcriptional gene regulation, a number of RNA-binding proteins have been found to play important roles in trypanosome biology [70]. For instance, overexpression of a single RNA-binding protein, RBP6, causes the spontaneous progression from procyclic forms to epimastigote and metacyclic forms in culture, mimicking the transitions occurring in the tsetse fly vector [71]. Another RNA-binding protein was found to be involved in maintenance of the bloodstream form developmental state, with RBP10 binding certain procyclic-specific transcripts and targeting them for repression [72]. Interestingly, regulatory RNA-binding proteins seem to dominate during the control of developmental gene expression as the parasite transitions between life-cycle stages; levels of constitutively expressed transcripts are, in contrast, apparently governed by codon usage bias [73, 74].

### A more nuanced picture of infection

Several developments have reshaped our view of the trypanosome infection dynamic and its interactions in a host context.

First, the perception of trypanosome infections as comprising cyclical ‘waves’ of parasitaemia in the bloodstream is now recognised as overly simplistic. Thus, rather than a procession of antigenic variants arising sequentially over time, it is now clear that there is considerable complexity in VSG expression dynamics, with many simultaneously expressed VSGs present within chronic infections albeit comprised of major and minor types [13, 39]. Chronic infections also do not involve an alternating fluctuation between mainly slender and mainly stumpy forms dependent on the overall parasitaemia, with instead, transmissible stumpy forms found to be prevalent throughout long-term infections in mice [75]. It was suggested that by limiting the number of slender forms, which can generate new antigenic variants, the parasites preserve their repertoire of available VSG types whilst also prolonging host survival and so maximizing the probability of transmission.

Secondly, we now understand how human infectivity is possible for some African trypanosomes. Subspecies of *T. brucei*, *T. b. gambiense* and *T. b. rhodesiense*, are the only African trypanosomes able to successfully establish in a human host, because other species are killed by the trypanolytic serum component, apolipoprotein L1 (APOL1). *Trypanosoma b. rhodesiense* has bypassed this defense through the evolution of Serum resistance associated (SRA) protein, derived from truncation of a VSG [76, 77]. The mechanisms by which *T. b. gambiense* outmaneuvers human defenses have taken longer to resolve but involve a role for *T. b. gambiense*-specific glycoprotein (TgsGP), deletion of which restored parasite sensitivity to human serum [78, 79]. Development of a recombinant *Papio papio* APOL1 mutant that could inhibit *T. b. gambiense* infection of mice illustrates how an increased knowledge of host-parasite interactions can open up new therapeutic possibilities [80].

Thirdly, the perception of trypanosomiasis as largely being a bloodstream infection has been revised. *Trypanosoma brucei*, for instance, can be found in abundance within adipose tissue. Adipose tissue parasites are transcriptionally distinct from those in the bloodstream, and may utilize fatty acids as a carbon source, indicating adaptation to their environment [81]. Pockets of *T. brucei* have also been found in the skin [22, 82]. The blood-brain barrier makes it challenging to effectively target parasites residing in the brain during drug treatment of late stage trypanosomiasis and parasites in the adipose tissue and skin may present a similar challenge for drug delivery. Drug efficacy may also be influenced by pharmacokinetic parameters which differ during the circadian cycle. Although hosts exhibit well defined circadian rhythms, trypanosome gene expression has also been shown to demonstrate circadian oscillation [83]. These oscillations can be observed in culture, following a period of entrainment, generating periodic peaks and troughs in the transcript levels of genes linked to metabolic processes. Interestingly, *T. brucei* infection can also disrupt the normal circadian rhythm of a murine host, leading to the proposition that sleeping sickness in humans represents a parasite-induced circadian disease [84].

## Three areas ripe for development

### Coherent assembly of signalling pathways and complexes

The ability of trypanosomes to sense and respond to changes in their environment is essential for their survival. However, the mechanisms underlying the transduction of extracellular signals is poorly understood, with most studies focusing on the function of individual molecules or molecular classes (e.g. kinases). Nonetheless, the development of high throughput technologies has allowed the analysis of signalling pathways at a genome-wide scale in trypanosomes. For example, in the QS response generating stumpy forms, components that fell into a potential hierarchical organisation were identified [61, 85]. To assemble these into a coherent pathway, a concerted knock-out and overexpression approach has been used, revealing dependencies between individual components [86]. With systematic gene ablation and mutation now possible *via* CRISPR/Cas9 [20], this approach can be extended to understand how components connect to transduce external cues. Refinements in transcriptome analysis, proteomics and phosphoproteomics can further enhance the resolution of signal network analysis and extend the understanding of the associated molecular interactions. Any external cue that generates a defined phenotypic response is accessible to dissection by an integration of these selection and analytical approaches, promising a more coherent understanding of how parasites interact with and respond to their environment to alter life history traits.

### The comparative biology of African trypanosome species

Although *T. brucei* has been the long-established model for African trypanosome biology, the reduced importance of human infections and the recognition of the distinct biology of *T. congolense* and *T. vivax* has generated renewed interest in these parasites. Research articles focused on *T. congolense* and *T. vivax* infections in the field have provided important insight into the dynamics of infection, host resistance and pathology, as well as prevalence of individual species in livestock, wild animals and tsetse flies. In contrast, knowledge of the molecular biology of *T. congolense* and *T. vivax* has lagged behind the wealth of information accumulated for *T. brucei*. The recent advances in culture techniques and genetic manipulation of *T. congolense* [87, 88] and *T. vivax* [89] will enable similar strides to be made in the understanding of the distinct cell biology of these important veterinary pathogens. Each African trypanosome species has developed significant and surprisingly distinct adaptations to their hosts, for example in their predicted surface-expressed proteins [90] and VSG gene archive and expression architecture [91, 92]. Each also shows a distinct developmental path in their tsetse fly vector [9]. It is now clear that we will not be able to fully rely on extrapolating information from *T. brucei* when it comes to investigating the biology of other trypanosome species.

### Host-parasite-vector interactions

Like other parasites, the fate of African trypanosomes is tied to interactions with their host, at least until they can escape to another host *via* their tsetse fly vector. Whilst immune evasion is one mechanism, trypanosomes also interact with the host immune system with the potential to regulate the course of infection, either through antibody clearance [93] or immune suppression [94]. These host-parasite interactions create the potential for genotype-by-genotype (G×G) interactions which can influence the virulence (parasite numbers) and pathology (disease) generated by parasites in the field. Virulence is often counterbalanced by the transmission benefits of infection chronicity, with the potential to generate evolutionary interactions between trypanosomes and their hosts. This is particularly complex for African trypanosomes which, unlike many parasites, have the ability to infect many mammalian host species, and can also exist in mixed coinfections with other trypanosome species. The tools and information to explore these interactions are emerging as different trypanosome species become genetically tractable and there is an expanding dataset of genomic sequences for field isolates. There is also a new focus on the interaction between the parasite and innate and acquired immune mechanisms [94, 95].

The passage of the trypanosome through the tsetse fly is also increasingly accessible, using sophisticated live cell microscopy [96], the development of labelled trypanosome lines which can be tracked during their journey in the insect vector [97] and the establishment of tools for the manipulation of tsetse gene expression using RNAi [98]. Exploring the tsetse-parasite interaction has also become tractable through increased sensitivity in transcriptome analyses using RNA-seq [99–101] and biochemical and proteomic analysis of the parasite metabolic pathways in the fly midgut [102–104]. All of these developments can also be deployed to understand the comparative transmission biology of different trypanosome species, given the distinct developmental paths of *T. brucei*, *T. congolense* and *T. vivax* through their vector [105]. Analyses of the interface between the trypanosome in the host and in their vector represent important studies because targeting parasite transmission remains the most effective and cost-efficient route to disease control [106].

Finally, the interaction between the trypanosome and its host in different compartments must be understood. The recent exciting discoveries of the novel niches exploited by trypanosomes in their mammalian host raises questions about the parasite’s biochemistry, drug sensitivity and immune susceptibility in these compartments, and their kinetics of exchange with the bloodstream population [107, 108]. These adaptations may also differ between different trypanosome species with consequences for drug efficacy in cattle populations with mixed infections, in particular.

## Conclusions

Trypanosomes continue to provide important insights to the evolution of eukaryotic cells, while remaining significant pathogens in sub-Saharan Africa. In combination, this makes the continued analysis of trypanosome biology compelling. With respect to fundamental biology, the unusual kinetochore composition in trypanosomatids has gained significant interest in the wider cell biology community, as has their unique mitochondrial biology and flagellar dynamics. Significant progress has also been made in therapeutics for these parasites, with several compounds at different stages of the drug development pipeline, most notably fexinidazole [109] and oxaboroles [110]. This has resulted from significant investment in the discovery of small molecule therapeutics which offer oral delivery and reduced toxicity. Similarly, the innovations in gene technology, including high throughput genetic screens, comprehensive protein localisation approaches ([111] http://tryptag.org) and the application of CRISPR technology to laboratory adapted and developmentally competent trypanosome lines have accelerated understanding of the parasite’s biology whilst helping the prediction of how drug resistance might emerge to new therapies. The first golden age of trypanosome biology was in the 1900s when the disease agent and vector was identified, and the second was in the 1980s and early 1990s where the unusual molecular mechanisms of trypanosomes were uncovered, particularly in relation to antigenic variation. We are currently in the third golden age, where this molecular understanding is applied to the fascinating biology of the parasite in the host and in the field, and for the discovery of new therapies.

## Additional file

**Additional file 1.** Poster on recent developments in the biology of African trypanosomes depicting the life-cycle of *Trypanosoma brucei*. In the left call out box are shown developments in the biology of trypanosomes in their mammalian host discussed in the text. In the right call out box are shown relevant features of the biology of trypanosomes in their arthropod vector, the tsetse fly. The bottom box highlights recent technological developments for dissecting gene function or location in trypanosomes.
