# Supplementary figures and images for: African trypanosomes
Source: Parasit Vectors. 2019 Apr 29;12:190. doi: 10.1186/s13071-019-3355-5 (PMC6489224; doi:10.1186/s13071-019-3355-5)

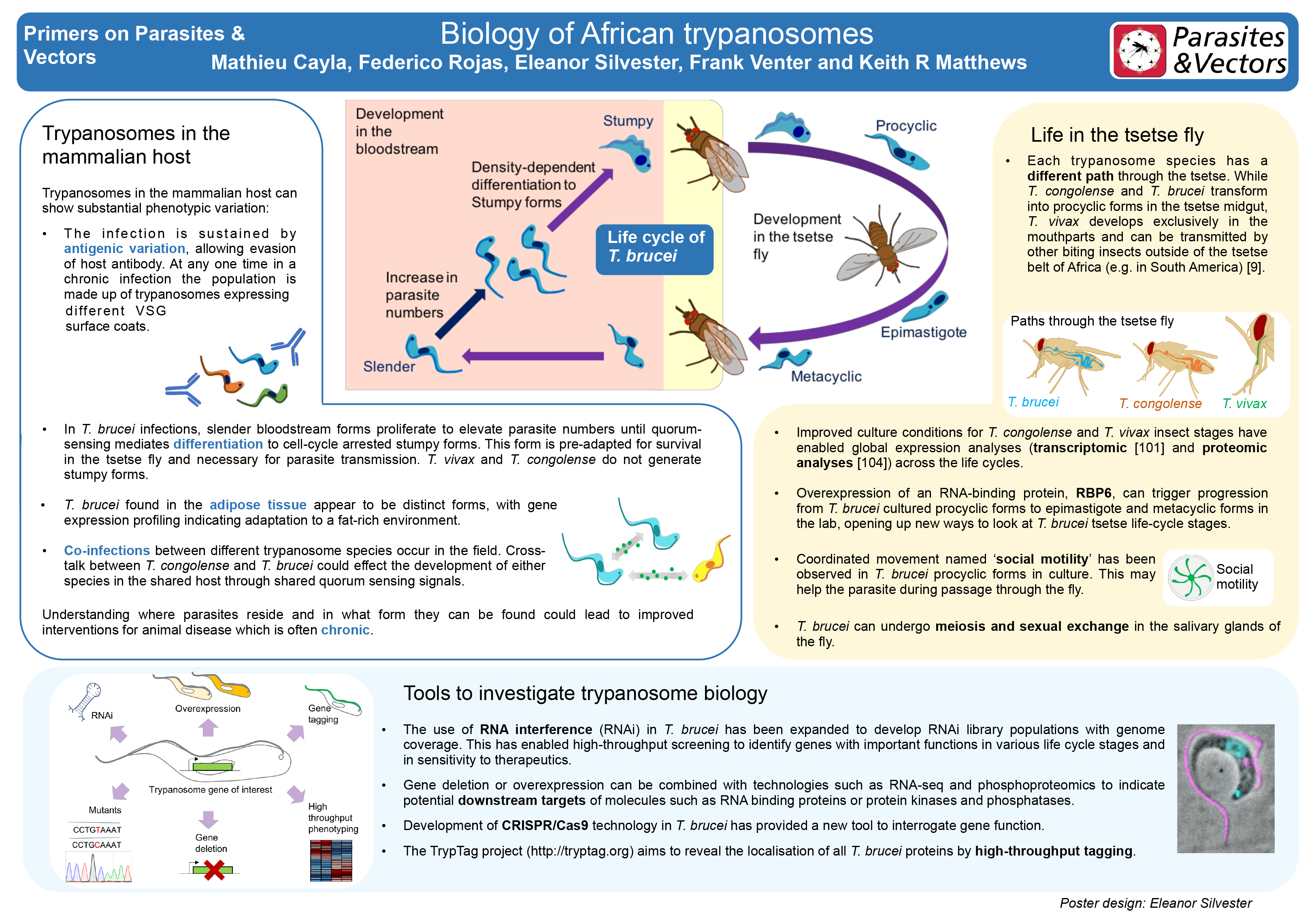

Supplement: Supplementary file 1 — Additional file 1. Poster on recent developments in the biology of African trypanosomes depicting the life-cycle of Trypanosoma brucei. In the left call out box are shown developments in the biology of trypanosomes in their mammalian host discussed in the text. In the right call out box are shown relevant features of the biology of trypanosomes in their arthropod vector, the tsetse fly. The bottom box highlights recent technological developments for dissecting gene function or location in trypanosomes. [file 13071_2019_3355_MOESM1_ESM.tif]
